# Genome Sequences of Listeria Phages Induced from Lysogenic Isolates of Listeria monocytogenes from Seafood and a Seafood Processing Environment in Thailand

**DOI:** 10.1128/genomeA.00546-18

**Published:** 2018-07-05

**Authors:** Hue Thi Kim Vu, Matthew J. Stasiewicz, Soottawat Benjakul, Kitiya Vongkamjan

**Affiliations:** a Department of Food Technology, Prince of Songkla University, Hat Yai, Thailand; b Department of Veterinary Hygiene and Food Safety, National Institute of Veterinary Research (NIVR), Dong Da, Hanoi, Vietnam; c Department of Food Science and Human Nutrition, University of Illinois Urbana-Champaign, Urbana, Illinois, USA

## Abstract

We report here the complete genome sequences of three Listeria phages (PSU-VKH-LP019, PSU-VKH-LP040, and PSU-VKH-LP041), which were newly induced from lysogenic isolates of Listeria monocytogenes from seafood and a seafood processing environment in Thailand. The three phages show circularly permuted double-stranded DNA genomes with sizes of 38.6, 39.6, and 48.3 kb.

## GENOME ANNOUNCEMENT

Prophage diversity is of interest since prophages are commonly present in the genomes of Listeria monocytogenes strains (1, 2). They play an important role in the evolution (3), survival, and persistence (4, 5) of L. monocytogenes. We have an ongoing project for screening lysogenic isolates of Listeria from various sources, including seafood and a seafood processing environment. Of these lysogenic isolates, an induced form of prophage(s) from selected isolates of L. monocytogenes was examined to understand the prophage diversity. We report here the complete genome sequences of three induced phages, PSU-VKH-LP019, PSU-VKH-LP040, and PSU-VKH-LP041 (hereafter referred to as LP019, LP040, and LP041, respectively).

Phage DNA was extracted by the phenol-chloroform method, as previously described (6). Fragmentation of DNA was performed, and high-quality sequencing libraries were sequenced using the Illumina HiSeq 2500 platform with 100-bp paired-end reads at Macrogen, Inc. (Seoul, South Korea). Then, low-quality reads were filtered by Trimmomatic (7). SOAP*denovo*2 was utilized for *de novo* assembly (8) before a prediction of open reading frames (ORFs) using Glimmer 2 (9) was made. An automatic genome annotation was performed by RAST (10) and Phaster (11) and then verified by BLAST (12), InterPro (http://www.ebi.ac.uk/interpro) (13), and Artemis (14). tRNA was detected using the tRNAscan-SE search server (15).

Sequencing of the induced phages by the Illumina HiSeq 2500 platform yielded 7 to 11 million reads, with an average sequencing coverage of 15,000×. *De novo* assembly resulted in a single contig for each phage, suggesting a complete genome. These genomes were circularly permuted terminally redundant double-stranded DNA genomes. The genome sizes of these phages ranged from 38 to 48 kb, which is consistent with the size range of previously reported temperate Listeria phages (16–19). A lysogeny module, including integrase and transcriptional regulator/repressor genes, was observed, thus confirming the temperate characteristic of these sequenced phages. No tRNAs were found in the genomes of these phages.

Phage LP019 was induced from a lysogenic L. monocytogenes isolate, PSU-KV-134LM, obtained from a seafood product (fish stick) using L. monocytogenes FSL J1-208 as a propagating host (20). This phage is 38,601 bp in length, with a GC content of 35.7%. A total of 66 ORFs were detected, of which 28 ORFs were assigned functions. Genome comparison by BLASTN of phage LP019 with the NCBI database showed 93% similarity with 62% sequence coverage to Listeria phage vB_LmoS_188, which was previously isolated from wild mushroom (21).

Two phages, LP040 and LP041, were induced from lysogenic L. monocytogenes isolate PSU-KV-036LM (from a seafood processing environment) using F2365 and FSL F2-695 as propagating hosts, respectively (H. T. K. Vu, S. Benjakula, and K. Vongkamjan, submitted for publication). Phage LP040 presents a genome size of 39,585 bp, with a GC content of 37.1%, whereas phage LP041 is 48,286 bp in length, with a GC content of 35.8%. For phage LP040, a total of 67 ORFs were detected, of which 32 ORFs were assigned functions. For phage LP041, a total of 81 ORFs were detected, but only 24 were assigned functions. Nucleotide sequence comparison by BLASTN revealed that LP040 showed a 91% similarity with 77% sequence coverage to Listeria phage vB_LmoS_293, isolated from mushroom compost (20). The genome of phage LP041 showed 96% similarity with 82% sequence coverage to phage B054 (16) previously induced from Listeria innocua WSLC 2054 (21).

### Accession number(s).

The genome sequences of these three induced Listeria phages, LP019, LP040, and LP041, have been deposited in GenBank under the accession no. MH341451, MH341452, and MH341453, respectively.
